# Epidemiology and Clinical Features of *Balamuthia mandrillaris* Infection, China

**DOI:** 10.3201/eid3207.251771

**Published:** 2026-07

**Authors:** Tzuyi Yang, Yan Lei, Xiaobo Feng, Zhirong Yao, Zhen Zhang

**Affiliations:** Xinhua Hospital, Shanghai Jiaotong University School of Medicine, Shanghai, China

**Keywords:** *Balamuthia mandrillaris*, parasites, China, epidemiology, encephalitis, granulomatous lesions

## Abstract

*Balamuthia mandrillaris* is a highly lethal free-living ameba that primarily affects the skin and central nervous system, manifesting clinically as chronic granulomatous lesions and granulomatous encephalitis. Once the central nervous system is involved, the mortality rate exceeds 90%. No standardized treatment regimen has yet been established. In this review, we summarized 66 cases reported from China. The median patient age was 36 years (range 10 months–77 years); 62.12% patients were male and 37.88% female. Fifty-five (83.33%) patients were immunocompetent. For 42 (63.64%) patients, initial symptoms were cutaneous manifestations; of those, central nervous system involvement subsequently developed in 25 (59.52%) patients. Twenty-four (36.36%) patients were hospitalized initially with encephalitis. Among the 63 patients with a known outcome, 43 (68.25%) succumbed to infection. For patients with cutaneous-only disease, the survival rate was 93.75%, whereas once the central nervous system was affected, mortality reached 96.00%.

*Balamuthia mandrillaris* is a free-living ameba classified within the phylum Amoebozoa, class Discosea, order Centramoebida, and family Balamuthiidae (*1*,*2*). *B. mandrillaris* are ubiquitously distributed in nature and exhibit 2 developmental stages: the trophozoite and the cyst. The trophozoite is 12–60 μm in diameter and is characterized by a round nucleus with a large, spherical, densely staining nucleolus. However, trophozoites with 2 or 3 nucleolar bodies have been seen, especially in infected tissues, which is a distinguishing feature in early diagnosis of *B. mandrillaris* infection (*3*). Trophozoites often display prominent spiny projections and pseudopodia. Under unfavorable environmental conditions, such as desiccation, extreme temperatures, or exposure to chemicals, trophozoites differentiate into cysts (*4*). The cysts are spherical, measuring 12–30 μm in diameter, with a single nucleus. The cyst wall appears double-layered under light microscopy and triple-layered under electron microscopy (*3*).

Previous studies have established that soil, water, and dust are the principal environmental reservoirs of *B. mandrillaris* and that the organism can enter the human body through breaches in the cutaneous barrier or through the lower respiratory tract (*5*). An additional report suggested that *B. mandrillaris* might invade the central nervous system through the olfactory nerve (*6*). Several case reports have also confirmed possible transmission through solid organ transplantation (*7*,*8*).

Globally, cases of *B. mandrillaris* infection have been reported on 5 continents, excluding just Africa and Antarctica. Most cases occur in hot, arid tropical and subtropical regions, particularly in the warm climate of the southwestern United States, tropical areas of Peru, and subtropical regions of China (*9*).

*B. mandrillaris* infection predominantly involves 2 organ systems in humans, the skin and the central nervous system (CNS). Cutaneous disease most frequently affects the nose, followed by the knees; other sites, such as the buttocks, thighs, arms, and chest, might also be involved. Lesions can be solitary or multifocal and usually manifest as asymptomatic, well-demarcated plaques with slightly raised annular borders; ulceration is uncommon (*3*). Lesion size ranges from 1 cm to several centimeters, and lesions typically have a reddish to dark red hue. The affected skin typically retains normal sensation and demonstrates a firm, rubbery, cartilaginous, or stone-like consistency on palpation (*10*). The cutaneous course is generally chronic, persisting for months to years. Without timely intervention, hematogenous dissemination to the CNS may occur (*4*). When the CNS is involved, the predominant clinical manifestation is granulomatous amebic encephalitis (GAE), also referred to as *B. mandrillaris* amebic encephalitis. The disease typically follows an acute to subacute course and manifests with nonspecific neurologic symptoms. Early features include headache, nausea, vomiting, fever, somnolence, gait disturbance, and impairments of consciousness and speech. When infection progresses rapidly, seizures, coma, and ultimately death can ensue (*3*,*10*). Once the CNS is affected, the mortality rate approaches 90% (*11*). Because of the nonspecific manifestation, CNS infection is often initially misdiagnosed as brain abscess, tumor, neurocysticercosis, or acute disseminated encephalomyelitis (*11*). *Balamuthia* appears capable of infecting both immunocompetent and immunocompromised persons. Previous reports in the United States have indicated that only 39% of patients were immunocompromised (*11*).

Given the rarity of this disease, the epidemiology and clinical features for *B. mandrillaris* infection in China remain incompletely characterized. To address this gap, we summarized all reported cases of *B. mandrillaris* infection in China, with the objective of delineating the epidemiology and clinical features.

## Methods

We conducted a comprehensive literature review to identify reported cases of *B. mandrillaris* infection in China. We included published articles in PubMed and the China National Knowledge Infrastructure through September 2025 using the following search terms: free-living amoebae, *Balamuthia mandrillaris*, infection, encephalitis, case report, diagnosis, treatment, and China. We conducted searches in English and Chinese. Two authors (T.Y. and Y.L.) independently reviewed the retrieved articles. Inclusion criteria were case reports or case series reporting >1 confirmed case of *B. mandrillaris* infection. We excluded duplicate publications and studies not involving *B. mandrillaris* infection. We extracted relevant data, such as patient characteristics, epidemiologic features, clinical manifestations, treatment strategies, and clinical outcomes. We reviewed 39 publications through September 2025 and identified 66 confirmed cases of *B. mandrillaris* infection in China beginning in 2002 (Appendix Table 1).

We classified the certainty of exposure sources on the basis of the descriptions provided in the literature. We defined the certainty of exposure sources as confirmed when a specific traumatic event or exposure was reported, the site of injury or exposure was consistent with the location of clinical manifestations, and the diagnosis was confirmed by histopathologic examination of the corresponding site. We considered the certainty of exposure sources probable when a specific traumatic event or exposure was described but the exact site of injury or exposure was not specified or when diagnosis was based on next-generation sequencing (NGS) only or histopathologic confirmation was not obtained from the corresponding site. We classified the certainty of exposure sources as possible when a suspected exposure was reported but lacked sufficient supporting evidence. The analyses in this study focused on confirmed and probable cases.

We also categorized the certainty of diagnosis on the basis of available evidence. We defined the certainty of diagnosis as confirmed when the diagnosis was established by histopathological examination but classified certainty of diagnosis diagnosed solely by NGS as probable, given that NGS alone can be insufficient to confirm a case because of the potential for cross-contamination. We calculated descriptive statistics and frequency distributions using SPSS Statistics for Windows version 17.0 (IBM, https://www.ibm.com).

## Results

### Epidemiology

We identified 39 articles from the literature search. All the included articles were case reports or case series. As of September 2025, the earliest reported case had disease onset in 2002; a total of 66 cases of *B. mandrillaris* infection in China have been reported in published English and Chinese language literature (Appendix Table 1).

Of the 66 cases, 41 (62.12%) patients were male and 25 (37.88%) female, yielding a male-to-female ratio of 1.64:1. Ages ranged from 10 months to 77 years (mean 36 years); 28 (42.42%) cases occurred in persons <18 years of age. Regarding occupational background, 8 (12.12%) patients were farmers, 1 was involved in animal husbandry, 1 was employed as a miner, and 1 worked in waste management. In the case of the remaining patients, 27 (40.91%) patients had no reported occupation and 28 (42.42%) were children <18 years of age. Among the 66 cases, only 23 had clearly reported dates of disease onset. Even within those 23 cases, we observed no apparent seasonal pattern of *B. mandrillaris* infection (Figure).

**Figure:**
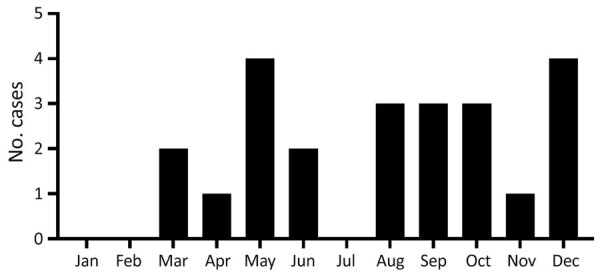
Monthly distribution of *Balamuthia mandrillaris* cases in study of epidemiology and clinical features of *B. mandrillaris* infection, China. Onset dates were specified in 23 case reports but unspecified in 43 others.

Cases were distributed across multiple provinces and municipalities in China. We determined distribution on the basis of clearly reported exposure locations; when such information was unavailable, we categorized cases according to the treatment location. Previous studies have faced similar limitations and therefore applied the same approach for case classification (*11*). Of the 66 cases, 38 cases had a clearly documented province of origin, whereas 28 were inferred from the treating hospital.

Among 66 cases, 55 (83.33%) patients were immunocompetent, 5 (7.58%) were immunocompromised or receiving immunosuppressive therapy (2 patients were receiving immunosuppressive therapy after kidney transplantation, 2 had type 2 diabetes mellitus, and 1 had breast cancer), and 2 (3.03%) had concomitant infections (1 patient had concurrent human herpesvirus 6 encephalitis and 1 had previous SARS-CoV-2 infection); the immune status of 4 (6.06%) patients was not specified. Among the 2 patients who received immunosuppressive therapy after kidney transplantation, 1 (case 52) underwent kidney transplantation 13 days before disease onset because of uremia and received tacrolimus, mycophenolate sodium enteric-coated tablets, methylprednisolone, and antithymocyteglobulin for antirejection therapy beginning on the first postoperative day (*12*). The other patient (case 57) had been on long-term immunosuppression for 14 years and was treated with cyclosporine, mycophenolate mofetil, and prednisone acetate; the patient also had a 2-month history of type 2 diabetes mellitus (*13*). The immunosuppressive therapy given to transplant recipients likely predisposes them to rapid progression of disease. Overall, 42 (63.64%) of 66 patients lived in rural areas, 3 (4.55%) lived in urban settings, and the remaining 21 (31.82%) had unspecified residence.

Regarding the certainty of transmission routes among the 66 cases, we classified 20 (30.30%) as confirmed, 4 (6.06%) as probable, 4 (6.06%) as possible, and 38 (57.58%) as unknown. Among the confirmed cases, 19 (95.83%) cases had a documented history of cutaneous trauma, and 1 (4.17%) case was linked to water exposure, in which previous nasal trauma followed by water contact resulted in nasal infection (*14*). Among the 4 probable cases, 2 had a documented history of skin trauma but without a clearly specified site corresponding to the location of clinical manifestations, and 2 had a documented history of skin trauma with a clearly identified site, but the diagnosis was established by NGS performed on specimens obtained from the brain. Among the 4 possible cases, 3 were suspected to be associated with water exposure: 1 patient had been exposed to rainwater 1 day before the onset of neurologic symptoms, 1 reported habitual consumption of untreated mountain spring water, and 1 was associated with accidental seawater aspiration. The remaining possible case-patient (case 52), who underwent kidney transplantation 13 days before disease onset, was classified as having a possible association with organ transplantation, given that donor-derived transmission and infection in other organ recipients from the same donor could not be confirmed (*12*). In contrast, case-patient 57, in whom infection developed 14 years after kidney transplantation, was considered less likely to have a transplant-related infection; the infection might instead have been associated with long-term immunosuppressive therapy, and the route of transmission remained unknown (*13*).

### Clinical Manifestations

Regarding clinical manifestations, 42 (63.64%) patients had initial symptoms of cutaneous lesions, most commonly plaques (85.71%), followed by scales (4.76%), erythema (2.38%), ulcers (2.38%), crusts (2.38%), and unspecified lesions (9.52%). Lesions were located on the face (78.57%), lower limbs (9.52%), upper limbs (7.14%), waist (2.38%), back (2.38%), or unspecified sites (2.38%). Among those 42 patients, CNS involvement subsequently occurred in 25 (59.52%) persons. For a total of 24 (36.36%) patients, initial manifestation was CNS disease, most frequently with headache (62.50%), fever (62.50%), vomiting (41.67%), gait instability or motor disturbance (29.17%), consciousness disturbance (25.00%), fatigue (20.83%), dizziness (16.67%), nausea (12.50%), speech disorders (12.50%), somnolence (8.33%), epilepsy (8.33%), blurred vision (4.17%), limb numbness (4.17%), personality or behavioral changes such as irritability (4.17%), delayed reaction (4.17%), and convulsions (4.17%). Among the 25 patients with both cutaneous and CNS involvement, the interval from the onset of skin lesions to neurologic manifestations ranged from 2 months to 9 years (mean 3.67 years); time interval was unspecified in 5 cases.

Regarding the certainty of relationship between clinical manifestations and transmission routes, among the 42 patients with cutaneous manifestations, 19 (45.24%) were classified as having a confirmed association with skin trauma. Four (9.52%) patients were classified as having a probable association with skin trauma, including 2 cases without a clearly specified site of exposure and 2 cases diagnosed by NGS of brain tissue. The remaining 19 (45.24%) patients had no detailed exposure information. Among the 24 patients who initially had CNS manifestations, 3 (12.50%) were classified as having a possible association with water exposure, and 1 (4.17%), who underwent transplantation 13 days before disease onset, was classified as having a possible association with organ transplantation (*12*). The remaining 20 (83.33%) had no documented exposure history.

### Diagnosis

Among the 66 reported cases, 38 (57.58%) were confirmed by histopathologic examination (with or without supporting NGS results). The remaining 28 (42.42%) were considered probable on the basis of NGS of brain tissue or cerebrospinal fluid only.

### Treatment

Among 66 reported patients, 44 (66.67%) received pharmacologic therapy, 9 (13.64%) underwent combined pharmacologic and surgical treatment, and 9 (13.64%) died before treatment could be initiated; for 4 (6.06%) patients, the treatment approach was unknown. For patients with cutaneous manifestations alone, treatment regimens have typically included >1 antibiotic from the lincosamide, macrolide, or tetracycline classes, administered with or without interferon and, in some cases, combined with surgical excision. For patients with CNS involvement, treatment regimens have generally consisted of surgical intervention (lesion resection or decompressive craniectomy) in combination with fluconazole, flucytosine, azithromycin (or tetracycline), and trimethoprim/sulfamethoxazole; metronidazole is added in some cases (Appendix Table 2).

### Outcome

Among 66 patients, outcome was known for 63 patients; 43 (68.25%) patients died and 20 (31.75%) survived (Appendix Table 1). Among 17 patients with cutaneous-only manifestations, 15 (93.75%) survived, 1 (6.25%) died from drug-induced liver failure after treatment with diminazene aceturate (*15*), and 1 had an unknown outcome. Among survivors, 11 received >1 lincosamide, macrolide, or tetracycline, with or without interferon and surgical excision.

Among 25 patients who initially demonstrated cutaneous manifestations and subsequently progressed to CNS involvement, 24 (96.00%) died and 1 (4.00%) survived. Of note, 11 of those patients received treatment regimens similar to those for patients demonstrating only cutaneous manifestations. However, although their skin lesions showed partial or marked improvement after treatment, all of them ultimately died after the development of neurologic symptoms.

Among 24 patients with CNS-only manifestations, 18 (81.82%) died, 4 (18.18%) survived, and 2 had unknown outcomes. Among 5 surviving patients with CNS involvement, with or without cutaneous lesions, 3 received combination treatment regimens consisting of fluconazole, flucytosine, azithromycin (or tetracyclines), and trimethoprim/sulfamethoxazole, with or without surgical excision; metronidazole was added in some cases. One patient underwent surgical excision followed by treatment with miltefosine, fluconazole, rifampin, albendazole, and amphotericin B; the treatment regimen for 1 patient was unknown. However, 2 patients who died received treatment regimens similar to those of the survivors, consisting of fluconazole, flucytosine, azithromycin, and trimethoprim/sulfamethoxazole, with or without surgical excision. Despite that treatment, their neurologic symptoms deteriorated and ultimately resulted in death. After CNS symptom onset, time to death ranged from 5 days to 6 months (mean 41.79 days); 24 patients (48.98%) died within 1 month.

## Discussion

In China, *B. mandrillaris* infections occur predominantly in men; most cases have been reported in northern regions and rural settings. A substantial proportion of affected persons are engaged in occupations such as farming, animal husbandry, and horticulture. The higher prevalence among men is likely attributable to their role as the primary labor force in rural areas, a pattern consistent with observations from the southwestern United States and Peru, where most infected patients were also male agricultural workers. Most cases have been reported in temperate climatic zones at latitudes comparable to those of the southwestern United States, consistent with environmental conditions favorable for the growth of *B. mandrillaris*.

In contrast to the United States, where ≈39% of reported cases have occurred in immunocompromised persons, most patients in China with *B. mandrillaris* infection have been immunocompetent; only 10.61% had underlying conditions such as hepatitis C, malignancy, viral infections, diabetes, uremia, or long-term immunosuppressive therapy. Those patients appear more likely to experience rapid progression to *B. mandrillaris* GAE, which might contribute to poor therapeutic outcomes. For example, in one case, a patient with a 2-year history of cutaneous lesions on the left knee underwent radical mastectomy for breast cancer. Although no postoperative immunosuppressive therapy was administered, the disease progressed rapidly to CNS involvement within 1 week of surgery and ultimately resulted in death (*16*).

In China, 63.64% of patients initially sought care for cutaneous manifestations, consistent with epidemiologic observations from Peru, where chronic granulomatous skin lesions also predominate. The lesions typically manifested as plaques, most frequently on the face, and many patients had a documented history of cutaneous trauma; lesions often developed at the site of injury. Those findings support the soil contact–skin injury hypothesis and align with the agricultural context of both China and Peru, where frequent soil exposure increases the risk for infection after traumatic skin injury. One reported case (case 52) was considered to have possibly acquired a donor-derived infection through hematogenous transmission through organ transplantation (*12*). A minority of cases have been possibly attributed to waterborne transmission; those patients had initial CNS symptoms such as headache, fever, and vomiting, resembling the clinical manifestations reported in the United States. A previous US study reported that 35 (85%) of 41 case-patients with documented soil exposure had engaged in soil-related activities, providing evidence supporting the soil contact–skin injury hypothesis. In addition, the same study also noted that 66% of patients in the United States who reported *B. mandrillaris* infection had a history of water exposure (*11*). In another previously reported case, infection with *B. mandrillaris* was associated with the use of nonsterile water for nasal lavage (*17*). In addition, another study reported that 57% of infected patients had exposure to stagnant water (*18*). Those reports suggest that water exposure might be a potential source of *B. mandrillaris* infection. Although the suspected water sources were not tested for *B. mandrillaris*, those findings indicate that water-related activities, such as swimming in ponds or rivers, might allow the organism to enter the body through breaches in the cutaneous barrier, through the nasal mucosa, or through the lower respiratory tract.

Patients initially demonstrating cutaneous disease achieved a survival rate of 93.75%. Most survivors received treatment regimens including 2 classes of antibiotics, with or without interferon, diminazene aceturate, or surgical excision. Diminazene aceturate, an antiprotozoal agent primarily used in veterinary medicine, has been reported in China to achieve favorable outcomes in the treatment of cutaneous *B. mandrillaris* infection. In those cases, the drug was used to reduce lesion size, often followed by surgical resection or adjunctive therapy (*15*). However, because of the lack of reported use in human treatment, available evidence is primarily derived from animal studies, in which its clinical application has been limited by potential adverse effects, including gastrointestinal symptoms (abdominal distension, nausea, and vomiting), cardiovascular manifestations (palpitations), hepatic and renal dysfunction (elevated liver enzymes and proteinuria), and severe neurotoxicity (myalgia, weakness, and polyneuritis) (*19*,*20*). Of note, among the 4 patients who were treated with diminazene aceturate reported in China, gastrointestinal and neurologic adverse effects developed in all patients, and 1 patient ultimately died of drug-related hepatic failure, underscoring concerns regarding its safety (*15*). Hepatotoxicity has not been reported with other commonly used therapeutic agents for this disease.

In cases where complete or near-complete surgical excision of intracranial lesions was achieved, surgery not only provided definitive confirmation of the infectious etiology but also might have contributed to disease management. In patients with rapidly increasing intracranial pressure because of extensive cerebral edema or space-occupying lesions, neurosurgical procedures such as decompressive craniectomy were used to relieve intracranial pressure, thereby affording critical time for pharmacologic therapy and host immune response. With regard to agents recommended by the US Centers for Disease Control and Prevention (such as miltefosine) and additional agents reported in the literature, such as pentamidine and nitroxoline, those drugs are not currently available in China. One patient survived after combined surgical and pharmacologic therapy with miltefosine, fluconazole, rifampin, albendazole, and amphotericin B (*21*). However, another patient succumbed to infection despite receiving a treatment regimen consisting of miltefosine, pentamidine, flucytosine, fluconazole, clarithromycin, amphotericin B, and trimethoprim/sulfamethoxazole (*16*).

Among the 66 cases of *B. mandrillaris* infection in mainland China summarized in this study, the survival rate was 93.75% for patients with cutaneous manifestations alone. However, once the disease progressed to CNS involvement, the mortality rate increased sharply to 96.00%, consistent with previous reports in other countries. In addition, when CNS symptoms were the initial manifestation, the mortality rate was as high as 81.82%. Of note, 11 patients still progressed to fatal neurologic involvement despite improvement in cutaneous manifestations after treatment, indicating that controlling cutaneous symptoms does not completely prevent neurologic involvement. It is likely owing to the limited ability of therapeutic agents to penetrate the blood–brain barrier.

An epidemiologic survey conducted in the United States reported that the mean interval from onset of CNS symptoms to death for *B. mandrillaris* GAE was 24 days (range 4–450 days; n = 43) (*11*). In contrast, among the cases from China included in this study, the mean interval from CNS symptom onset to death was 39.45 days (range 7–180 days; n = 33), and 48.98% of patients died within 1 month of CNS symptom onset. Those findings underscore the extremely poor prognosis of *Balamuthia mandrillaris* infection once the CNS becomes involved.

With advances in diagnostic technology and improved detection capacity, the number of confirmed cases of *B. mandrillaris* infection in China has gradually increased, underscoring the need for heightened clinical awareness. Given the exceedingly high mortality rate associated with this disease, further in-depth research is urgently needed to enable earlier prevention and more timely detection, with the ultimate goal of reducing both the incidence and mortality of this devastating infection. When patients display suspicious epidemiologic exposures or cutaneous manifestations, *B. mandrillaris* infection should be considered in the differential diagnosis. 

Appendix Additional information about the epidemiology and clinical features of *Balamuthia mandrillaris* infection, China
