# Supplementary material for: Epidemiology and Clinical Features of Balamuthia mandrillaris Infection, China
Source: Emerg Infect Dis. 2026 Jul;32(7):1058–64. doi: 10.3201/eid3207.251771 (PMC13322445; doi:10.3201/eid3207.251771)
Supplement: Appendix — Additional information about the epidemiology and clinical features of Balamuthia mandrillaris infection, China [file 25-1771-Techapp-s1.pdf]

Article DOI: https://doi.org/10.3201/eid3207.251771

EID cannot ensure accessibility for supplementary materials supplied by authors. Readers who have difficulty accessing supplementary content should contact the authors for assistance.

Epidemiology and Clinical Features of *Balamuthia mandrillaris* Infection, China

Appendix

Appendix Table 1. Summary of 66 Cases of *Balamuthia mandrillaris* Infection in China.

Cases	Age (Years) / Sex	Distribution	Occupation	Exact date of symptom onset	Immunocompromised conditions	Route of transmission (The certainty)	Causes	Skin symptoms	Brain symptoms	Diagnosis (The certainty)	Treatments	Final outcomes as of publication
1 (*1*)	49/ Male	Distributed among 12 Provinces of People's Republic of China.	Unknown	Unknown	None	Cutaneous trauma (confirmed)	Trauma to right eyebrow, temple, and jaw angle after a fall.	Red plaques on right eyebrow, temple, and angle of jaw.	None	Skin biopsy(confirmed)	The cutaneous lesions disappeared after treatment with lincomycin, but the skin lesions relapsed twice.	Free of disease for 18 y.
2 (*1*)	6/ Male		None	Unknown	None	Cutaneous trauma (confirmed)	Trauma to the right temple.	Red plaque on right temple, diameter 7 cm.	Developed encephalitis after 2 y. Presented with headache and fever.	Skin biopsy(confirmed)	Died before treatment.	Died of disease.(Died on days 5 after the onset of clinical symptoms.)
3 (*1*)	13/ Female		None	Unknown	None	Cutaneous trauma (confirmed)	Dog bite to the left face.	Red plaque involving the whole left side of the face.	Developed encephalitis after 5 y. Presented with epilepsy and apathy.	Skin biopsy(confirmed)	The cutaneous lesions regressed prominently after treatment with lincomycin and azithromycin, whereas the encephalitis evolved after treatment.	Died of disease.(Died on months 6 after the onset of clinical symptoms.)
4 (*1*)	8/ Male		None	Unknown	None	Unknown	Unknown	Red plaque on the left side of the face, diameter 7 cm.	Developed encephalitis after 3 y. Presented with fever, headache,	Skin biopsy(confirmed)	Lincomycin, azithromycin, interferon-α, and interleukin-2 were ineffective.	Died of disease.(Died on week 1 after the onset of clinical symptoms.)

Cases	Age (Years) / Sex	Distribution	Occupation	Exact date of symptom onset	Immunocompromised conditions	Route of transmission (The certainty)	Causes	Skin symptoms	Brain symptoms	Diagnosis (The certainty)	Treatments	Final outcomes as of publication
									somnolence, and coma.			
5 (*1*)	5/ Male		None	Unknown	None	Unknown	Unknown	Red plaque on the nose and right side of the face, diameter 7 cm.	Developed encephalitis after 2 y. Presented with headache and fever.	Skin biopsy(confirmed)	Died before treatment.	Died of disease.(Died on week 1 after the onset of clinical symptoms.)
6 (*1*)	18/ Female		None	Unknown	None	Cutaneous trauma (confirmed)	Trauma to the left face.	Red macule/plaque involving the whole left side of the face and nose.	Developed encephalitis after 9 y. Presented with headache, somnolence, and unable to walk.	Skin biopsy(confirmed)	The cutaneous lesion regressed after treatment with lincomycin and interferon-γ in the beginning but relapsed after 2 mo.	Died of disease.(Died on months 2 after the onset of clinical symptoms.)
7 (*1*)	20/ Male		Unknown	Unknown	None	Cutaneous trauma (confirmed)	Brick-related trauma to left face.	Red plaque on the left side of the face with focal scales, diameter 9 cm.	Developed encephalitis after 2 y. Presented with apathy, anisocoria, and attenuation of light reflection.	Skin biopsy(confirmed)	Lincomycin and azithromycin were ineffective.	Died of disease.(Died on months 1·5 after the onset of clinical symptoms.)
8 (*1*)	7/ Female		None	Unknown	None	Cutaneous trauma (probable)	Trauma during play with other children.	Red plaque on the nose and the surrounding skin, diameter 5 cm.	None. However, neuroimaging revealed a focal infectious lesion in the left occipito-parietal lobe.	Skin biopsy(confirmed)	The cutaneous lesion regressed after treatment with lincomycin and interferon-γ, while the brain infection regressed very slowly with the medicine. Thereafter, it was surgically excised.	Free of disease for 13 y.
9 (*1*)	7/ Female		None	Unknown	None	Cutaneous trauma (confirmed)	Fall-related facial trauma after being struck by a car.	Red plaque on the central face, the edge was not clear, diameter larger than 10 cm.	Developed encephalitis after 3 y. Presented with headache and fever.	Skin biopsy(confirmed)	The cutaneous lesions showed partial regression after treatment with lincomycin and interferon-γ for 4 mo. Following discontinuation of therapy, the lesions enlarged and progressed to encephalitis.	Died of disease.(Died on month 1 after the onset of clinical symptoms.)

Cases	Age (Years) / Sex	Distribution	Occupation	Exact date of symptom onset	Immunocompromised conditions	Route of transmission (The certainty)	Causes	Skin symptoms	Brain symptoms	Diagnosis (The certainty)	Treatments	Final outcomes as of publication
10 (*1*)	74/ Male		Unknown	Unknown	None	Cutaneous trauma (confirmed)	Trauma to the left face.	Red plaque on the left side of the face, diameter 10 cm.	Developed encephalitis after 3 y. Presented with gait instability, tremor, somnolence, and incontinence.	Skin biopsy(confirmed)	The cutaneous lesions showed partial regression after treatment with lincomycin and interferon-γ for 2 mo. Following discontinuation of therapy, the lesions enlarged and progressed to encephalitis.	Died of disease.(Died on months 2 after the onset of clinical symptoms.)
11 (*1*)	21/ Male		Unknown	Unknown	None	Unknown	Unknown	Red plaque on nose and surrounding skin, diameter 7 cm.	Developed encephalitis after 2 y. Presented with headache, somnolence, and coma.	Skin biopsy(confirmed)	Itraconazole, rifampin, isoniazid, and ethambutol were ineffective. Lincomycin and interferon-γ were ineffective.	Died of disease.(Died on month 1 after the onset of clinical symptoms.)
12 (*1*)	61/ Female		Unknown	Unknown	None	Cutaneous trauma (confirmed)	Trauma to the left face after a fall.	Red plaque on left side of the face, diameter 10 cm.	None	Skin biopsy(confirmed)	The cutaneous lesion showed regression after treatment with lincomycin, doxycycline, and interferon-γ.	Free of disease for 13 y.
13 (*1*)	3/ Male		None	Unknown	None	Cutaneous trauma (confirmed)	Trauma to the chin after a fall.	Red plaque on chin, diameter 4.5 cm.	None	Skin biopsy(confirmed)	The patient underwent surgery, and followed by six months of treatment with lincomycin and interferon-γ.	Free of disease for 12 y.
14 (*1*)	68/ Female		Unknown	Unknown	None	Unknown	Unknown	Three plaques around a surgical scar. The largest diameter ≈2 cm.	None	Skin biopsy(confirmed)	The patient underwent surgery but relapsed. Consequently, therapy with rifampin, isoniazid, and ethambutol was administered	Lost to follow-up.
15 (*1*)	13/ Male		None	Unknown	None	Cutaneous trauma (confirmed)	Trauma to the right earlobe.	Plaque on the right ear and the surrounding skin, diameter 8 cm.	Developed encephalitis after 5 y. Presented with somnolence, speech disorders, and diplopia.	Skin biopsy(confirmed)	The cutaneous lesion regressed after treatment with lincomycin, azithromycin, and interferon-γ, whereas	Died of disease.(Died on month 1 after the onset of clinical symptoms.)

Cases	Age (Years) / Sex	Distribution	Occupation	Exact date of symptom onset	Immunocompromised conditions	Route of transmission (The certainty)	Causes	Skin symptoms	Brain symptoms	Diagnosis (The certainty)	Treatments	Final outcomes as of publication
											encephalitis evolved after treatment.	
16 (*1*)	5/ Male		None	Unknown	None	Unknown	Unknown	Red plaque on nose and surrounding skin, diameter of 10 cm.	Developed encephalitis after 3.5 y. Presented with left eye movement restriction, blurred vision, right arm muscle weakness, and inability to stand and walk.	Skin biopsy(confirmed)	lincomycin, interferon-γ, doxycycline, and rifampin were ineffective. Azithromycin showed improvement at first but was ineffective after 2 mo.	Died of disease.(Died on days 15 after the onset of clinical symptoms.)
17 (*1*)	39/ Male		Unknown	Unknown	None	Cutaneous trauma (confirmed)	Trauma to the right face after a fall from a bicycle.	Red plaque on right side of the face and two solitary lesions on arm and waist.	None	Skin biopsy(confirmed)	The cutaneous lesion was cured after 6 mo of treatment with lincomycin and interferon-γ.	Free of disease for 10 y.
18 (*1*)	9/ Male		None	Unknown	None	Unknown	Unknown	Red plaque on nose, diameter 3 cm.	None	Skin biopsy(confirmed)	The cutaneous lesion was cured with clindamycin and topical mupirocin for 3 mo.	Free of disease for 9 y.
19 (*1*)	22/ Female		Unknown	Unknown	None	Unknown	Recent upper respiratory infection.	Red plaque on left nasolabial fold and nose, diameter 4 cm.	Developed encephalitis after 6 mo. Presented with headache, dizziness, vomiting, and epilepsy.	Skin biopsy(confirmed)	Lincomycin, azithromycin and interferon-α were ineffective.	Died of disease.(Died on months 2 after the onset of clinical symptoms.)
20 (*1*)	58/ Female		Unknown	Unknown	None	Cutaneous trauma (confirmed)	Trauma to the right ear after a fall.	Dark red plaque on the right ear and the surrounding skin, diameter 8 cm.	None	Skin biopsy(confirmed)	The cutaneous lesion was cured after 1 y of treatment with rifampin, isoniazid, and ethambutol.	Free of disease for 8 y.
21 (*1*)	7/ Male		None	Unknown	None	Cutaneous trauma (confirmed)	Facial trauma after a fall.	Red plaque on the nose and face, diameter 8 cm.	Developed encephalitis after 8 y. Unable to walk, evolved into somnolence, and coma.	Skin biopsy(confirmed)	The cutaneous lesion showed partial regression after initiation of treatment with lincomycin, interferon-γ, and	Died of disease.(Died on weeks 2 after the onset of clinical symptoms.)

Cases	Age (Years) / Sex	Distribution	Occupation	Exact date of symptom onset	Immunocompromised conditions	Route of transmission (The certainty)	Causes	Skin symptoms	Brain symptoms	Diagnosis (The certainty)	Treatments	Final outcomes as of publication
											azithromycin, but relapsed after 3 mo.	
22 (*1*)	48/ Male		Unknown	Unknown	None	Cutaneous trauma (confirmed)	Nasal trauma after a fall.	Red plaque on the nose and surrounding skin, diameter 10 cm.	None	Skin biopsy(confirmed)	The cutaneous lesion was cured after treatment with lincomycin, interferon-γ, and azithromycin for 5 mo.	Free of disease for 5 y.
23 (*1*)	4/ Female		None	Unknown	None	Cutaneous trauma (confirmed)	Trauma to the left zygomatic region after a fall.	Red plaque on left zygomatic region, diameter 5 cm.	None	Skin biopsy(confirmed)	The patient underwent surgery, and followed by lincomycin and interferon-γ treatment.	Free of disease for 5 y.
24 (*1*)	4/ Male		None	Unknown	None	Cutaneous trauma (confirmed)	Trauma to the left face after striking an iron gate.	Red plaque on left side of the face, diameter 12 cm.	Developed encephalitis after 4.5 y. Presenting with somnolence and coma.	Skin biopsy(confirmed)	The cutaneous lesion showed partial regression after treatment with lincomycin and interferon-γ but was ineffective after 6 mo.	Died of disease.(Died on month 1 after the onset of clinical symptoms.)
25 (*1*)	57/ Female		Unknown	Unknown	None	Unknown	Unknown	Red plaque on right side of the face, diameter 10 cm.	None	Skin biopsy(confirmed)	The cutaneous lesion was cured after treatment with lincomycin and interferon-γ for 3 mo.	Free of disease for 4 y.
26 (*1*)	51/ Male		Unknown	Unknown	None	Cutaneous trauma (probable)	Trauma after a fall following alcohol consumption .	Red plaque on the forehead, diameter 7 cm.	None	Skin biopsy(confirmed)	lincomycin, interferon-γ, and azithromycin were ineffective. Thereafter, the patient underwent surgery and treated with lincomycin, interferon-γ, and azithromycin for 6 mo.	Free of disease for 4 y.
27 (*1*)	15/ Male		None	Unknown	None	Unknown	Unknown	Red plaque around the nose, diameter 6 cm.	None	Skin biopsy(confirmed)	The cutaneous lesions showed partial regression after treatment with lincomycin, interferon-γ, and azithromycin. Six months later the patient underwent	Free of disease for 3 y.

Cases	Age (Years) / Sex	Distribution	Occupation	Exact date of symptom onset	Immunocompromised conditions	Route of transmission (The certainty)	Causes	Skin symptoms	Brain symptoms	Diagnosis (The certainty)	Treatments	Final outcomes as of publication
											surgery and treated with lincomycin, interferon-γ, and azithromycin for 8 mo.	
28 (*1*)	69/ Male		Unknown	Unknown	None	Unknown	Unknown	Red plaque on the forehead, diameter 7 cm.	Developed encephalitis after 4 y. Presenting with somnolence, coma, and inability to stand and walk.	Skin biopsy(confirmed)	The cutaneous lesion showed slight regress after treatment with lincomycin and interferon-γ. However, recurrence occurred after treatment was discontinued.	Died of disease.(Died on months 2 after the onset of clinical symptoms.)
29 (*2*)	9/ Female	Jiangxi (treatment location)	None	Unknown	None	Unknown	Unknown	None	Presented with consciousness disturbance, fever, headache, and vomiting.	CSF NGS(probable)	Died before treatment.	Died of disease.(Died on weeks 3 after the onset of clinical symptoms.)
30 (*3*)	9/ Female	Jiangxi (treatment location)	None	12 December 2018	None	Unknown	Unknown	None	Presented with vomiting, fever, headache, motor disturbance, and consciousness disturbance.	CSF NGS(probable)	On the day of admission, cefotaxime and acyclovir were administered for anti-infection. On the third day, isoniazid, rifampin, pyrazinamide, ethambutol, and metronidazole were added. The patient died on the ninth day of admission. However, the specific doses of the relevant drugs were not reported.	Died of disease.(Died within 1 mo after the onset of clinical symptoms.)
31 (*4*)	13/ Female	Fujian (exposure location)	None	Skin:March 2017 Nervous system:March 2019	None	Unknown	Unknown	Multiple red plaque on the left thigh.	Developed encephalitis after 2 y. Presented with dizziness, blurred vision, and vomiting.	Skin biopsy and CSF NGS(confirmed)	Subsequent treatment with Albendazole(400 mg, po, QD)and azithromycin(10 mg/kg/day, iv, QD),liposomal amphotericin B(0.1–0.5–1–1.5–2–2.5	Died of disease.(Died months 2 after being diagnosed with BAE.)

Cases	Age (Years) / Sex	Distribution	Occupation	Exact date of symptom onset	Immunocompromised conditions	Route of transmission (The certainty)	Causes	Skin symptoms	Brain symptoms	Diagnosis (The certainty)	Treatments	Final outcomes as of publication
											mg/kg/day, iv, QD), 5-fluorocytosine(100 mg/kg/day, iv, TID), TMP–SMZ(72 mg/kg/day, po, TID)were effective. However, cardiovascular adverse effects developed. The regimen was therefore changed to liposomal amphotericin B, 5-fluorocytosine, and fluconazole(400 mg, iv, QD) which were effective, but relapse eventually occurred.	
32 (*5*)	2/ Male	Gansu (treatment location)	None	Unknown	None	Unknown	Unknown	None	Presented with fever and vomiting.	Brain tissue NGS(probable)	Died before treatment.	Died of disease.(Died on months 2 after the onset of clinical symptoms.)
33 (*6*)	69/ Male	Hebei (treatment location)	Farmer	Unknown	Concomitant infections: HHV-6	Unknown	Unknown	None	Presented with headache, fever, fatigue, speech disorders, and irritability.	Brain tissue NGS(probable)	Liposomal amphotericin B liposomes(initially 7mg, increased to 24.5mg on the second day, then increased to 42mg on the 13th day and maintained at this level for 29 d)was effective, but treatment was discontinued due to cardiovascular adverse events.	Died of disease.(Died on months 2 after the onset of clinical symptoms.)
34 (*7*)	37/ Female	Zhejiang (exposure location)	Unknown	Unknown	Immunocompromised: radical mastectomy for left breast cancer 1 week earlier.	Cutaneous trauma (probable)	Trauma to the left knee near a garbage dump 2 y earlier.	Dark red plaque on the left knee, measuring 15cm×10cm.	Presented with dizziness, vomiting, nausea, and somnolence.	Brain tissue NGS(probable)	Amphotericin B (40 mg, po, QD, 18d), flucytosine(500 mg, po, TID, 18d), fluconazole (800 mg, po, QD, 18d), TMP–SMZ(1.44 g, po, QID, 18d),	Died of disease.(Died on days 27 post-admission.)

Cases	Age (Years) / Sex	Distribution	Occupation	Exact date of symptom onset	Immunocompromised conditions	Route of transmission (The certainty)	Causes	Skin symptoms	Brain symptoms	Diagnosis (The certainty)	Treatments	Final outcomes as of publication
											clarithromycin(500 mg, po, QD, 18d), pentamidine(300 mg, po, QD, 18d), and miltefosine(50 mg, po, TID, 11d) were ineffective.	
35 (*8*,*9*)	15/ Male	Guizhou (exposure location)	None	May 2019	None	Water exposure (confirmed)	Swimming in a natural pond with nasal trauma.	Red plaque on the nose	Developed encephalitis after 2 mo. Presenting with fever.	Skin biopsy, Skin tissue NGS, and Brain tissue NGS(confirmed)	Amphotericin B (15mg, iv, QD, 5d), fluconazole (200mg, iv, QD, 5d), Sulfamethoxazole (800mg, po, QID, 5d), Azithromycin (0.5g, iv, QD, 5d), Fluocytosine (1.5g, po, TID, 5d).	Died of disease. (Died weeks 2 after being diagnosed with BAE.)
36 (*10*)	54/ Male	Guangxi (treatment location)	Unknown	November 2020	None	Unknown	Unknown	None	Numbness and fatigue in the left limbs.	CSF NGS and Brain biopsy(confirmed)	The patient underwent neurosurgical intervention combined with TMP–SMZ (0.96 g, po, BID, 50d), azithromycin (0.5g, po, QD, 50d), flucytosine(2.5g, iv, bid, 50d), and amphotericin B(increased gradually with 5–10–15–20–25–30mg, iv, qd, 10d). However, two weeks later, a recurrence occurred at another site. A second surgery, together with TMP–SMZ, azithromycin, flucytosine, and fluconazole (0.6 g, iv, QD, 40d), led to improvement, after which oral SMZ and azithromycin were continued during follow-up.	Free of disease.

Cases	Age (Years) / Sex	Distribution	Occupation	Exact date of symptom onset	Immunocompromised conditions	Route of transmission (The certainty)	Causes	Skin symptoms	Brain symptoms	Diagnosis (The certainty)	Treatments	Final outcomes as of publication
37 (*11*)	54/ Male	Guangdong (exposure location)	Unknown	August 2020	None	Unknown	Unknown	Plaque on the medial aspect of the right knee and posterior aspect of the right thigh.	Presented with headache, dizziness, and twitching of his right limb.	Brain tissue NGS(probable)	The patient died after 2 d of treatment with albendazole(0.4g, po, BID),metronidazole(0.6g, iv, Q8H), voriconazole(0.2g, iv, Q12H)and fluconazole(0.4g, iv, QD).	Died of disease.(Died on days 15 after the onset of clinical symptoms.)
38 (*12*)	7/ Male	Anhui (treatment location)	None	December 2013	None	Cutaneous trauma (probable)	Trauma to the forehead after a fall.	Red plaque on the forehead, with some scales on the surface.	Developed encephalitis after 6 y. Presented with fever, headache, vomiting, gait instability, and somnolence.	CSF NGS(probable)	Died before treatment. Previous empirical treatment of skin lesions with recombinant human interferon-α2b (3 million international units, twice per week) for 1 mo was ineffective.	Died of disease.(Died on month 1 after the onset of clinical symptoms.)
39 (*13*)	14/ Male	Shaanxi (treatment location)	None	December 2015	Unknown	Unknown	Unknown	Red plaque on nose, measuring 10cm×7cm.	None	Skin biopsy(confirmed)	D1–310: Interferon γ, 1–2 million unit, ih, QOD; linconmycin, 600mg, im, BID. D311–619: The patient consulted another hospital and was treated with traditional Chinese herb medicine for ≈10 mo. D620–624: The patient was rediagnosed as cutaneous Balamuthia mandrillaris infection. D625–637: Day 625, diminazene aceturate, 270mg (5mg/kg) (108mg, local injection; 162mg, iv). Day 628, diminazene aceturate, 270mg,	Free of disease.

Cases	Age (Years) / Sex	Distribution	Occupation	Exact date of symptom onset	Immunocompromised conditions	Route of transmission (The certainty)	Causes	Skin symptoms	Brain symptoms	Diagnosis (The certainty)	Treatments	Final outcomes as of publication
											iv. Day 631, diminazene aceturate, 270mg, iv. Day 633, diminazene aceturate, 270mg (90mg, local injection; 180mg, iv). Day 634–637, diminazene aceturate, 270mg, iv, QD. D638–753: Day 639–690, albendazole, 400mg, po, BID. Day 691–753, albendazole, 400mg, po, BID; itraconazole, 200mg, po, BID. D754–770: Diminazene aceturate, 135mg, iv, BID, for 9 times. Albendazole, 800mg, po, BID; itraconazole, 200mg, po, BID. D771–913: Albendazole, 800mg, po, BID; itraconazole, 200mg, po, BID. Day914–1018: Albendazole, 800mg, po, BID. D1018–2050: No treatment.	
40 (*13*)	57/ Female	Shaanxi (treatment location)	Unknown	August 2017	Unknown	Unknown	Unknown	Red plaque on lower left back, measuring 17cm×7cm.	None	Skin biopsy(confirmed)	D1–23: Day 1–10, Diminazene aceturate, 180mg (3mg/kg), iv, QOD. Day 19–23, Diminazene aceturate, 300mg (5mg/kg), iv, QD. D24–64: Hepatoprotective	Died of drug-induced liver failure.

Cases	Age (Years) / Sex	Distribution	Occupation	Exact date of symptom onset	Immunocompromised conditions	Route of transmission (The certainty)	Causes	Skin symptoms	Brain symptoms	Diagnosis (The certainty)	Treatments	Final outcomes as of publication
											therapy was given to the patient. During this period artificial liver support system was used for 7 times. D65: The patient died of liver failure, hepatic encephalopathy, metabolic acidosis and subsequent hypotensive shock, respiratory and circulatory failure.	
41 (*13*)	9/ Male	Shaanxi (treatment location)	None	October 2019	Unknown	Cutaneous trauma (confirmed)	Trauma to the right face after a fall 1 y earlier.	2 red plaques on the right side of face, measuring 8cm×7cm and 3cm×2cm.	None	Skin biopsy(confirmed)	D1–9: Diminazene aceturate, 66mg (3mg/kg), Iv, QOD, for 5 times. D10–24: No treatment. D25–37: Diminazene aceturate, 110mg (5mg/kg), iv, QOD, for 7 times. D38–59: Albendazole, 200mg, po, QD, from day 45 to day 59. Albendazole was stopped due to loss of appetite from day 60. D60–76: Diminazene aceturate, 110mg (5mg/kg), iv, QOD, for 9 times. D77–193: Lincomycin, 600mg, im, BID from day 91 to day 147, TID from day 148 to day 193. Clindamycin, 600mg, iv, QD from day 131 to day 193. Albendazole,	Free of disease.

Cases	Age (Years) / Sex	Distribution	Occupation	Exact date of symptom onset	Immunocompromised conditions	Route of transmission (The certainty)	Causes	Skin symptoms	Brain symptoms	Diagnosis (The certainty)	Treatments	Final outcomes as of publication
											200mg, po, QD from day 131 to day 193. Topical imiquimod, QOD from day 97 to day 104, QD from day 105 to day 133. Imiquimod was stopped because of slight erosion of the skin. D194–202: Diminazene aceturate, 110mg (5mg/kg), iv, QOD, for 5 times. D203–383: Lincomycin, 900mg, im or iv, QD. D384–723: No treatment.	
42 (*13*)	61/ Male	Shaanxi (treatment location)	Unknown	September 2019	Unknown	Cutaneous trauma (confirmed)	Trauma caused by a steel bar.	Red plaque on nose, measuring 5cm×4cm.	None	Skin biopsy(confirmed)	D1–7: Diminazene aceturate, 320mg (5mg/kg), iv, QOD, for 4 times. D8–13: No treatment. D14: Surgical excision of the lesion with 0.5cm free edge. D14–21: Lincomycin, 1200mg, im, QD. D22–24: Day 22, diminazene aceturate, 320mg (5mg/kg), iv. Day 24, diminazene aceturate, 320mg (5mg/kg), IV. Lincomycin, 1200mg, im, QD. D25–70: Lincomycin, 1200mg, im, QD. D70: Cosmetic reconstruction with skin dilation to repair	Free of disease.

Cases	Age (Years) / Sex	Distribution	Occupation	Exact date of symptom onset	Immunocompromised conditions	Route of transmission (The certainty)	Causes	Skin symptoms	Brain symptoms	Diagnosis (The certainty)	Treatments	Final outcomes as of publication
											the ulceration and scar. D71–194: Lincomycin, 1200mg, im, QD, for 6 mo. D195–364: No treatment.	
43 (*14*)	56/ Male	Zhejiang (treatment location)	Employment in waste management	Unknown	Immunocompromised: type 2 diabetes mellitus.	Unknown	Unknown	Dark red rashes on the left and right forearms, measuring 10cm×5cm and 1cm×1cm.	Presented with nausea, vomiting, stiff neck, and coma.	Brain tissue NGS(probable)	Albendazole (400 mg, QD, po, 7d) and fluconazole (600 mg, iv, QD, 7d) were ineffective.	Died of disease.(Died on month 1 after the onset of clinical symptoms.)
44 (*15*)	53/ Male	Yunnan (treatment location)	Unknown	May 2022	None	Unknown	Unknown	Scabs on the back.	Presented with headache, fever, vomiting, and consciousness disturbance.	CSF NGS(probable)	Piperacillin–tazobactam(3g, iv, Q8H, 2d), fluconazole(0.4g, iv, QD, 2d), and levornidazole(1g, iv, QD, 2d))were ineffective.	Died of disease.
45 (*16*)	61/ Male	Jiangxi (exposure location)	Poultry farmer	August 2021	None	Unknown	Unknown	None	Presented with headache.	CSF NGS and Brain biopsy(confirmed)	Following neurosurgery, the patient was treated with azithromycin, fluconazole, flucytosine, and TMP–SMZ for 4 weeks, during which neurologic symptoms improved; however, deterioration occurred in the fifth week, and the patient subsequently died.	Died of disease. (Died weeks 2 after brain surgery.)
46 (*17*)	64/ Female	Yunnan (exposure location)	Farmer	June 2020	None	Unknown	Unknown	None	Presented with dizziness, headache, nausea, vomiting, and gait instability.	CSF NGS(probable)	Died before treatment.	Died of disease.(Died on days 22 after the onset of clinical symptoms.)
47 (*18*, *19*)	49/ Female	Hebei (treatment location)	Farmer	Unknown	None	Cutaneous trauma (confirmed)	Trauma to the right lower limb.	Dark red rashes on the right lower limb.	Developed encephalitis after 6 mo.	Brain tissue NGS and Brain biopsy(confirmed)	After decompressive craniectomy, treatment with	Free of disease.

Cases	Age (Years) / Sex	Distribution	Occupation	Exact date of symptom onset	Immunocompromised conditions	Route of transmission (The certainty)	Causes	Skin symptoms	Brain symptoms	Diagnosis (The certainty)	Treatments	Final outcomes as of publication
									Presented with headache, dizziness, nausea, and vomiting.		azithromycin, (500mg, po, QD, 25d), metronidazole (0.5g, iv, Q8H, 25d), flucytosine (1.5g, iv, Q6H, 25d), fluconazole (0.4g, iv, QD, 25d), and TMP–SMZ (1.6g, po, Q6H, 25d),was effective	
48 (*20*)	66/ Female	Anhui (treatment location)	Farmer	March 2023	None	Unknown	Unknown	None	Presented with headache.	CSF NGS(probable)	Unknown(the patient's family chose to forgo further treatment)	Died of disease.
49 (*21*)	77/ Female	Shandong (treatment location)	Unknown	Unknown	None	Unknown	Unknown	None	Presented with dizziness, consciousness disturbance, and delayed reaction.	CSF NGS(probable)	Died before treatment.	Died of disease.(Died within 1 mo after the onset of clinical symptoms.)
50 (*22*)	77/ Female	Shandong (exposure location)	Unknown	Unknown	None	Unknown	Unknown	None	Presented with dizziness and gait instability.	CSF NGS(probable)	Unknown(the patient's family chose to forgo further treatment)	Died of disease.(Died within 1 mo after the onset of clinical symptoms.)
51 (*23*)	72/ Female	Shandong (treatment location)	Farmer	September 2023	None	Unknown	Unknown	None	Presented with fever, headache, and vomiting.	CSF NGS(probable)	Tinidazole(200ml, iv, QD, 2d) was ineffective. In contrast, fluconazole(400mg, iv, QD), metronidazole(0.44 g, po, TID), flucytosine,(0.75 g, po, FID) minocycline(100mg, po, BID), and sulfamethoxazole(1.44 g, po, TID) were effective.	Free of disease
52 (*24*)	47/ Male	Guangxi (treatment location)	Unknown	October 2022	Immunosuppressive therapy (following kidney transplantation for uremia)	Organ transplantation (possible)	Kidney transplantation 13 d before onset.	None	Presented with fever.	Brain tissue NGS(probable)	Liposomal amphotericin B, sodium sulfadiazine, Sulperazon, linezolid, fluconazole, azithromycin,	Free of disease.

Cases	Age (Years) / Sex	Distribution	Occupation	Exact date of symptom onset	Immunocompromised conditions	Route of transmission (The certainty)	Causes	Skin symptoms	Brain symptoms	Diagnosis (The certainty)	Treatments	Final outcomes as of publication
											flucytosine, albendazole tablets	
53 (*25*)	62/ Male	Gansu (treatment location)	Farmer	May 2021	None	Unknown	Unknown	None	Presented with headache, dizziness, nausea, blurred vision, and fatigue.	CSF NGS(probable)	Treatment with metronidazole (0.5g, iv, Q8H) led to slight improvement after 3 d, but the condition subsequently worsened.	Died of disease.(Died on months 4 after the onset of clinical symptoms.)
54 (*26*)	52/ Male	Hunan (treatment location)	Unknown	Unknown	None	None	Unknown	None	Presented with headache, gait instability, and consciousness disturbance.	Brain tissue NGS(probable)	Died before treatment.	Died of disease.
55 (*27*)	58/ Male	Guangdong (exposure location)	Unknown	Unknown	None	Water exposure (possible)	Exposure to rainfall 1 d before symptom onset.	None	Presented with headache, fever, and stiff neck.	CSF NGS and Brain biopsy(confirmed)	Ceftriaxone(4g, iv, QD), fluconazole(0.6g, iv, QD), liposomal amphotericin B(started at an initial dose of 5mg, iv, QD, gradually increasing to 40mg, iv, QD), SMZ(0.96 g, po, Q8H), Metronidazole (0.8g, po, TID) were ineffective; subsequent ventricular drainage surgery(left-sided lateral external ventricular drain (LEVD)+ endoscopic third ventriculostomy (ETV)+ intraventricular lavage) was also ineffective.	Died of disease.(Died on days 33 after the onset of clinical symptoms.)
56 (*28*)	76/ Female	Guangdong (treatment location)	Unknown	Unknown	None	Unknown	Unknown	None	Presented with headache, fever, vomiting, and fatigue.	CSF NGS(probable)	Treatment with azithromycin, albendazole, and fluconazole for 6 d resulted in clinical improvement.	Unknown
57 (*29*)	61/ Male	Henan (treatment location)	Mushroom Cultivation Industry	May 2022	Immunocompromised: 14-y post–renal transplantation with	Unknown	Unknown	None	Presented with fever, dizziness, gait	CSF NGS(probable)	The patient died after 1 d of treatment with metronidazole,	Died of disease.(Died on weeks 2 after the

Cases	Age (Years) / Sex	Distribution	Occupation	Exact date of symptom onset	Immunocompromised conditions	Route of transmission (The certainty)	Causes	Skin symptoms	Brain symptoms	Diagnosis (The certainty)	Treatments	Final outcomes as of publication
					immunosuppressive therapy; type 2 diabetes mellitus.				instability, and somnolence.		sulfadiazine, azithromycin, and ketoconazole.	onset of clinical symptoms.)
58 (*30*)	66/ Female	Anhui (treatment location)	Unknown	Unknown	None	Unknown	Unknown	None	Presented with headache.	CSF NGS(probable)	Unknown	Died of disease.
59 (*31*)	6/ Male	Guizhou (treatment location)	None	June 2023	None	Water exposure (possible)	Seawater aspiration at a Hainan beach 3 weeks prior.	None	Presented with fever, vomiting, consciousness disturbance, convulsions, and movement disorders.	CSF NGS(probable)	Treatment with azithromycin, fluconazole, and SMZ was associated with elevated transaminase levels compared with baseline, and the therapeutic effect was uncertain.	Unknown
60 (*32*)	77/ Female	Jiangsu (treatment location)	Farmer	April 2024	None	Unknown	Unknown	Purple rash on the left dorsum of hand.	Presented with fever, dizziness, vomiting, and delayed reaction.	CSF NGS(probable)	Rifampin and ornidazole were ineffective.	Died of disease.(Died on days 9 after the onset of clinical symptoms.)
61 (*33*)	73/ Male	Hunan (exposure location)	Unknown	September 2023	Immunocompromised: type 2 diabetes mellitus.	Water exposure (possible)	Habitual consumption of mountain spring water.	None	Presented with fever, headache, vomiting, fatigue, and speech disorders.	CSF NGS(probable)	Albendazole(400mg, po, Q12H), flucytosine(1g, po, Q6H), TMP–SMZ(1.44 g, po, Q6H), and fluconazole(400mg, iv, QD) were ineffective.	Died of disease.(Died months 5 after being diagnosed with BAE.)
62 (*34*)	9/ Female	Jiangxi (treatment location)	None	December 2023	None	Unknown	Unknown	None	Presented with fever, headache, vomiting, consciousness disturbance, and somnolence.	CSF NGS(probable)	Died before treatment.	Died of disease.(Died on days 30 after the onset of clinical symptoms.)
63 (*35*)	3/ Male	Anhui (treatment location)	None	October 2023	None	Unknown	Unknown	Dark red rash on the right temporal region, measuring 3cm×5cm.	Developed encephalitis after 1 y. Presented with fever, lower limb weakness, and	CSF NGS(probable)	Amphotericin B and fluconazole for 8 d were ineffective.	Died of disease.(Died on month 1 after the onset of clinical symptoms.)

Cases	Age (Years) / Sex	Distribution	Occupation	Exact date of symptom onset	Immunocompromised conditions	Route of transmission (The certainty)	Causes	Skin symptoms	Brain symptoms	Diagnosis (The certainty)	Treatments	Final outcomes as of publication
									consciousness disturbance.			
64 (*36*, *37*)	35/ Male	Beijing (treatment location)	Miner	Unknown	None	Unknown	Unknown	None	Presented with fever, headache, nausea, and vomiting.	Brain tissue NGS(probable)	Meropenem, azithromycin, TMP-SMZ, and fluconazole were ineffective.	Died of disease.(Died on months 2 after the onset of clinical symptoms.)
65 (*38*)	3/ Male	Zhejiang (treatment location)	None	Unknown	None	None	Unknown	None	Presented with fever, speech disorders, and epilepsy.	Brain tissue NGS(probable)	After surgical excision, treatment with miltefosine, fluconazole, rifampin, albendazole, and amphotericin B for 1 mo was effective.	Free of disease.
66 (*39*)	10-mo/Male	Xinjiang (exposure location)	None	Unknown	Concomitant infections: SARS-CoV-2 infection 2 weeks before symptom onset.	None	Unknown	None	Presented with fever and epilepsy.	Brain tissue NGS(probable)	Azithromycin, albendazole, TMP–SMZ, fluconazole, and flucytosine were ineffective.	Died of disease.(Died on 1.5 mo after the onset of clinical symptoms.)

*CSF, cerebrospinal fluid; NGS, next-generation sequencing; TMP–SMZ, trimethoprim–sulfamethoxazole; BAE, *Balamuthia mandrillaris* amebic encephalitis; SMZ, sulfamethoxazole.

Appendix Table 2. Treatment regimens of 66 cases of *Balamuthia mandrillaris* infection in People's Republic of China

System involvement	Treatments	The dose/route of treatments	The duration of treatments	Therapeutic effect	Outcomes/ Cause of death	The timing of the reported outcomes	No. cases
Only cutaneous involvement	Lincosamides and interferon.	Unknown	Unknown	The cutaneous lesions subsided.	Survival	Unknown	2 (case17,25)
	Lincosamides.	Unknown	Unknown	In one case, cutaneous lesions subsided but recurred twice; in another case, the cutaneous lesions resolved completely.	Survival	Unknown	2 (case1,18)
	Surgical excision combined with lincosamides, macrolides, and interferon.	Unknown	Unknown	Excision of cutaneous lesions without recurrence.	Survival	Unknown	2 (cas26,27)
	Surgical excision combined with lincosamides and interferon.	Unknown	Unknown	Excision of cutaneous lesions without recurrence.	Survival	Unknown	2(case13,23)
	Lincosamides and macrolides.	Unknown	Unknown	The cutaneous lesions subsided.	Survival	Unknown	1(case8)
	Lincosamides, macrolides, and interferon.	Unknown	Unknown	The cutaneous lesions subsided.	Survival	Unknown	1(case22)
	Lincosamides, tetracyclines, and interferon.	Unknown	Unknown	The cutaneous lesions subsided.	Survival	Unknown	1(case12)
	Rifampin, isoniazid, and ethambutol.	Unknown	Unknown	The cutaneous lesions subsided.	Survival	Unknown	1(case20)
	After treatment with diminazene aceturate, albendazole and itraconazole were administered.	Diminazene aceturate:270mg (5mg/kg) iv qod ×1w→270mg (5mg/kg) iv qd ×1w<br>Itraconazole:200mg, po, BID<br>Albendazole:400mg, po, BID	Diminazene aceturate:2 weeks<br>Itraconazole:22 d<br>Albendazole:1379 d	After treatment with diminazene aceturate, the cutaneous lesions subsided.	Survival	1430 d	1(case39)
	After treatment with diminazene aceturate, the residual skin lesions were surgically excised.	Diminazene aceturate, 66mg (3mg/kg), Iv, QOD, for 26 times<br>Albendazole:200mg, po, QD<br>Lincomycin: 600mg, im, BID×56d→TID 45d→900mg, im or iv, QD×180d<br>Clindamycin:600mg, iv, QD<br>Topical imiquimod: QOD ×7d→QD ×28d	Diminazene aceturate:52 d<br>Albendazole:77 d<br>Lincomycin:281 d<br>Clindamycin:62 d<br>Topical imiquimod:35 d	After treatment with diminazene aceturate, the cutaneous lesions markedly subsided, and the residual lesions were excised without recurrence.	Survival	723 d	1(case41)
	After treatment with diminazene aceturate, the residual skin lesions were surgically excised, followed by administration of lincosamides.	Diminazene aceturate, 320mg (5mg/kg), iv, QOD, for 6 times<br>Lincomycin:1200mg, im, QD<br>Surgical excision of the lesion with 0.5cm free edge	Diminazene aceturate:12 d<br>Lincomycin:180 d	After treatment with diminazene aceturate, the cutaneous lesions markedly subsided, and the residual lesions were excised without recurrence.	Survival	1 y	1(case42)
	First: Lincosamides and interferon.<br>Second: Diminazene aceturate.	Diminazene aceturate, 180mg (3mg/kg), iv, QOD for 5 times→300mg (5mg/kg), iv, QD for 5 times	Diminazene aceturate:15d	First: No improvement in cutaneous lesions.<br>Second: The cutaneous lesions subsided.	Death(Drug-induced liver failure due to diminazene aceturate treatment)	64 d	1(case40)
	First: Surgery.	Unknown	Unknown	First: Recurrence of cutaneous lesions.	Unknown	Unknown	1(case14)

System involvement	Treatments	The dose/route of treatments	The duration of treatments	Therapeutic effect	Outcomes/ Cause of death	The timing of the reported outcomes	No. cases
	Second: Rifampin, isoniazid, and ethambutol.			Second: unknown			
Concurrent cutaneous and CNS involvement	Decompressive craniectomy combined with metronidazole, fluconazole, flucytosine, azithromycin, and TMP–SMZ.	Azithromycin:500mg, po, QD<br>Metronidazole:0.5g, iv, Q8H<br>Flucytosine:1.5g, iv, Q6H<br>Fluconazole:0.4g, iv, QD<br>TMP–SMZ:1.6g, po, Q6H	Azithromycin:25 d<br>Metronidazole:25 d<br>Flucytosine: 25 d<br>Fluconazole: 25 d<br>TMP–SMZ:25 d	Neurologic symptoms improved.	Survival	25 d	1(case47)
	Lincosamides and interferon.	Unknown	Unknown	Resolution of cutaneous lesions, followed by recurrence and progression to encephalitis.	Death	Unknown	5(case6,9,10,24,28)
	Lincosamides, macrolides, and interferon.	Unknown	Unknown	Marked resolution of cutaneous lesions, with no improvement in neurologic symptoms.	Death	Unknown	3(case15,19,21)
	Lincosamides and macrolides.	Unknown	Unknown	Marked resolution of skin lesions, with no improvement in neurologic symptoms.	Death	Unknown	2(case3,7)
	Lincosamides, macrolides, interferon and interleukin-2 (IL-2).	Unknown	Unknown	No improvement in neurologic symptoms.	Death	Unknown	1(case4)
	First: itraconazole, rifampin, isoniazid, and ethambutol.<br>Second: lincosamides and interferon.	Unknown	Unknown	First: no improvement in neurologic symptoms.<br>Second: no improvement in neurologic symptoms.	Death	Unknown	1(case11)
	First: lincosamides, tetracyclines, interferon, and rifampin.<br>Second: macrolides.	Unknown	Unknown	First: no improvement in neurologic symptoms.<br>Second: no improvement in neurologic symptoms.	Death	Unknown	1(case16)
	Miltefosine, pentamidine, flucytosine, fluconazole, clarithromycin, amphotericin B, and TMP–SMZ.	Amphotericin B:40 mg, po, QD<br>Flucytosine:500 mg, po, TID<br>Fluconazole:800 mg, po, QD<br>TMP–SMZ:1.44 g, po, QID<br>Clarithromycin:500 mg, po, QD<br>Pentamidine:300 mg, po, QD<br>Miltefosine:50 mg, po, TID	Amphotericin B: 18 d<br>Flucytosine:18 d<br>Fluconazole:18 d<br>TMP–SMZ:18 d<br>Clarithromycin: 18 d<br>Pentamidine:18d<br>Miltefosine:11 d	No improvement in neurologic symptoms.	Death	27 d	1(case34)
	First: lincosamides, tetracyclines, and itraconazole.<br>Second: itraconazole and linezolid.<br>Third: fluconazole, flucytosine, macrolides, and SMZ.	Unknown	2 weeks	First: no improvement in neurologic symptoms.<br>Second: no improvement in neurologic symptoms.<br>Third: no improvement in neurologic symptoms.	Death	2 weeks	1(case35)
	First: albendazole, macrolides, and TMP-SMZ.<br>Second: albendazole and fluconazole.	Albendazole:400 mg, po, QD<br>Azithromycin:10 mg/kg/day, iv, QD<br>Liposomal amphotericin B:0.1–0.5–1–1.5–2–2.5 mg/kg/day, iv, QD	Unknown	First: no improvement in neurologic symptoms.<br>Second: no improvement in neurologic symptoms.	Death	2 mo	1(case31)

System involvement	Treatments	The dose/route of treatments	The duration of treatments	Therapeutic effect	Outcomes/ Cause of death	The timing of the reported outcomes	No. cases
	Third: liposomal amphotericin B, flucytosine, and TMP-SMZ.<br>Fourth: amphotericin B, flucytosine, and fluconazole.	5-Fluorocytosine:100 mg/kg/day, iv, TID<br>TMP–SMZ:72 mg/kg/day, po, TID<br>Fluconazole:400 mg, iv, QD		Third: cardiovascular adverse reactions occurred (chest tightness and ventricular fibrillation).<br>Fourth: no improvement in neurologic symptoms.			
	Amphotericin B and fluconazole.	Unknown	Amphotericin B: 8 d<br>Fluconazole: 8 d	No improvement in neurologic symptoms.	Death	8 d	1(case63)
	Albendazole, metronidazole, and fluconazole.	Albendazole:0.4g, po, BID<br>Metronidazole:0.6g, iv, Q8H<br>Fluconazole:0.4g, iv, QD	Albendazole:2 d<br>Metronidazole:2 d<br>Fluconazole:2 d	No improvement in neurologic symptoms.	Death	2 d	1(case37)
	Albendazole and fluconazole.	Albendazole:400 mg, QD, po<br>Fluconazole:600 mg, iv, QD	Albendazole:7 d<br>Fluconazole:7 d	No improvement in neurologic symptoms.	Death	7 d	1(case43)
	Piperacillin–tazobactam, fluconazole, and ornidazole.	Piperacillin–tazobactam:3g, iv, Q8H<br>Fluconazole:0.4g, iv, QD<br>Ornidazole:1g, iv, QD	Piperacillin–tazobactam: 2 d<br>Fluconazole:2 d<br>Ornidazole: 2 d	No improvement in neurologic symptoms.	Death	2 d	1(case44)
	Rifampin and ornidazole.	Unknown	Unknown	No improvement in neurologic symptoms.	Death	9 d	1(case60)
	Died before treatment	Unknown	Unknown	None	Death	Unknown	3(case2,5,38)
Only CNS involvement.	First: tinidazole.<br>Second: metronidazole, fluconazole, flucytosine, minocycline, and TMP–SMZ.	Tinidazole:200ml, iv, QD<br>Fluconazole:400mg, iv, QD<br>Metronidazole:0.44 g, po, TID<br>Flucytosine:0.75 g, po, FID<br>Minocycline:100mg, po, BID<br>Sulfamethoxazole:1.44 g, po, TID	Unknown	First: no improvement in neurologic symptoms.<br>Second: Neurologic symptoms resolved.	Survival	Unknown	1(case51)
	First: surgical excision combined with amphotericin B, flucytosine, azithromycin, and TMP–SMZ.<br>Second: surgical excision combined with fluconazole, flucytosine, azithromycin, and TMP–SMZ.	TMP–SMZ:0.96 g, po, BID<br>Azithromycin:0.5g, po, QD<br>Flucytosine:2.5g, iv, bid, 50d<br>Amphotericin B: increased gradually with 5–10–15–20–25–30mg, iv, qd<br>Fluconazole:0.6 g, iv, QD	TMP–SMZ:50 d<br>Azithromycin:50 d<br>Flucytosine:50 d<br>Amphotericin B:10 d<br>Fluconazole:40 d	After the first surgical excision, neurologic symptoms resolved, but the lesions recurred on the contralateral side; after the second surgery, neurologic symptoms resolved again.	Survival	50 d	1(case36)
	After surgical excision, treatment with miltefosine, fluconazole, rifampin, albendazole, and amphotericin B.	Unknown	1 mo	Neurologic symptoms resolved.	Survival	1 mo	1(case65)
	Unknown	Unknown	Unknown	Unknown	Survival	Unknown	1(case52)
	Metronidazole.	Case30: Unknown<br>Case53: Metronidazole 0.5g, iv, Q8H	Case30: Unknown<br>Case53: Metronidazole 3 d	No improvement in neurologic symptoms.	Death	Case30: Unknown<br>Case53: 3 d	2(case30,53)
	Surgical excision combined with fluconazole, flucytosine, azithromycin, and TMP-SMZ.	Unknown	Unknown	Neurologic symptoms initially improved, but deteriorated abruptly after the fifth week.	Death	2 weeks	1(case45)
	First: ceftriaxone, fluconazole, flucytosine,	Ceftriaxone:4g, iv, QD<br>Fluconazole:0.6g, iv, QD	Unknown	First: no improvement in neurologic symptoms.	Death	Unknown	1(case55)

System involvement	Treatments	The dose/route of treatments	The duration of treatments	Therapeutic effect	Outcomes/ Cause of death	The timing of the reported outcomes	No. cases
	amphotericin B, metronidazole, and SMZ. Second: ventricular drainage surgery.	Liposomal amphotericin B: started at an initial dose of 5mg, iv, QD, gradually increasing to 40mg, iv, QD SMZ:0.96 g, po, Q8H Metronidazole:0.8g, po, TID		Second: no improvement in neurologic symptoms.			
	Metronidazole, sulfadiazine, azithromycin, and ketoconazole.	Unknown	Unknown	No improvement in neurologic symptoms.	Death	1 d	1(case57)
	Albendazole, azithromycin, TMP–SMZ, fluconazole, and flucytosine.	Unknown	Unknown	No improvement in neurologic symptoms.	Death	Unknown	1(case66)
	Albendazole, fluconazole, flucytosine, and TMP-SMZ.	Albendazole:400mg, po, Q12H Flucytosine:1g, po, Q6H TMP–SMZ:1.44 g, po, Q6H Fluconazole:400mg, iv, QD	Unknown	No improvement in neurologic symptoms.	Death	5 mo	1(case61)
	Albendazole, azithromycin, and fluconazole.	Unknown	6 d	Neurologic symptoms improved after 6 d of treatment.	Unknown	Unknown	1(case56)
	Azithromycin, fluconazole, and SMZ.	Unknown	Unknown	Unknown	Unknown	Unknown	1(case59)
	Meropenem, azithromycin, TMP-SMZ, and fluconazole.	Unknown	Unknown	No improvement in neurologic symptoms.	Death	Unknown	1(case64)
	Liposomal amphotericin B.	Initially 7mg, increased to 24.5mg on the second day, increased to 42mg on the 13th day and maintained at this level for 29 d	29 d	Neurologic symptoms improved, but treatment was discontinued due to cardiovascular adverse reactions (elevated cardiac enzymes and troponin levels).	Death	29 d	1(case33)
	Died before treatment	Unknown	Unknown	None	Death	Unknown	6(case29,32,46,49,54,62)
	Unknown	Unknown	Unknown	Unknown	Death	Unknown	3(case48,50,58)

*TMP–SMZ, trimethoprim–sulfamethoxazole; SMZ, sulfamethoxazole.

References

1. Wang L, Cheng W, Li B, Jian Z, Qi X, Sun D, et al. *Balamuthia mandrillaris* infection in China: a retrospective report of 28 cases. Emerg Microbes Infect. 2020;9:2348–57. PubMed https://doi.org/10.1080/22221751.2020.1835447

2. Yi Z, Zhong J, Wu H, Li X, Chen Y, Chen H, et al. *Balamuthia mandrillaris* encephalitis in a child: case report and literature review. Diagn Microbiol Infect Dis. 2021;100:115180. PubMed https://doi.org/10.1016/j.diagmicrobio.2020.115180

3. Wang P, Zhong J, Yu X, Wu H, Yi Z, Zha J, et al. *Balamuthia mandrillaris* encephalitis: a case report and literature review [in Chinese]. Journal of Nanchang University:Medical Sciences. 2020;60:104–7.

4. Wu X, Yan G, Han S, Ye Y, Cheng X, Gong H, et al. Diagnosing *Balamuthia mandrillaris* encephalitis via next-generation sequencing in a 13-year-old girl. Emerg Microbes Infect. 2020;9:1379–87. PubMed https://doi.org/10.1080/22221751.2020.1775130

5. Yang Y, Hu X, Min L, Dong X, Guan Y. *Balamuthia mandrillaris*-related primary amoebic encephalitis in China diagnosed by next generation sequencing and a review of the literature. Lab Med. 2020;51:e20–6. PubMed

6. Yuan D, Jiang H, Xiao B, Liu L, Wen Y, Jiang J, et al. *Balamuthia mandrillaris* combined with Human herpes virus 6A encephalitis [in Chinese]. Journal of Brain and Nervous Diseases. 2020;28:345–9.

7. Hu J, Zhang Y, Yu Y, Yu H, Guo S, Shi D, et al. Encephalomyelitis caused by *Balamuthia mandrillaris* in a woman with breast cancer: a case report and review of the literature. Front Immunol. 2022;12:768065. PubMed https://doi.org/10.3389/fimmu.2021.768065

8. Ai J, Zhang H, Yu S, Li J, Chen S, Zhang W, et al. A case of fatal amoebic encephalitis caused by *Balamuthia mandrillaris*, China. Infect Genet Evol. 2022;97:105190. PubMed https://doi.org/10.1016/j.meegid.2021.105190

9. Tao K, Wang T, Zhang L, Yang XC, Zhai ZF. Fatal *Balamuthia mandrillaris* infection with red plaques on the nasal dorsum as the first presentation. An Bras Dermatol. 2022;97:498–500. PubMed https://doi.org/10.1016/j.abd.2021.12.001

10. Peng L, Zhou Q, Wu Y, Cao X, Lv Z, Su M, et al. A patient with granulomatous amoebic encephalitis caused by *Balamuthia mandrillaris* survived with two excisions and medication. BMC Infect Dis. 2022;22:54. PubMed https://doi.org/10.1186/s12879-021-07020-8

11. Xu C, Wu X, Tan M, Wang D, Wang S, Wu Y. Subacute *Balamuthia mandrillaris* encephalitis in an immunocompetent patient diagnosed by next-generation sequencing. J Int Med Res. 2022;50:3000605221093217. PubMed https://doi.org/10.1177/03000605221093217

12. Zhang Z, Liang J, Wei R, Feng X, Wang L, Wang L, et al. Facial *Balamuthia mandrillaris* infection with neurological involvement in an immunocompetent child. Lancet Infect Dis. 2022;22:e93–100. PubMed https://doi.org/10.1016/S1473-3099(21)00334-0

13. Wang L, Li B, Zhao T, Wang L, Jian Z, Cheng W, et al. Treatment of cutaneous *Balamuthia mandrillaris* infection with diminazene aceturate: a report of 4 cases. Clin Infect Dis. 2022;75:1637–40. PubMed https://doi.org/10.1093/cid/ciac356

14. Fan X, Chen T, Yang H, Gao Y, Chen Y. Encephalomyelomeningitis caused by *Balamuthia mandrillaris*: a case report and literature review. Infect Drug Resist. 2023;16:727–33. PubMed https://doi.org/10.2147/IDR.S400692

15. Wang Q, Zhou W, Wang Z, Tang J, Fu K, Liu S, et al. *Balamuthia mandrillaris* encephalitis causing brain herniation: a case report and literature review [in Chinese]. Chinese Journal of Critical Care Medicine. 2023;43:76–9.

16. Liu J, Zhang W, Wu S, Zeng T, Luo F, Jiang Q, et al. A clinical case report of *Balamuthia* granulomatous amoebic encephalitis in a non-immunocompromised patient and literature review. BMC Infect Dis. 2023;23:245. PubMed https://doi.org/10.1186/s12879-023-08228-6

17. Yao S, Chen X, Qian L, Sun S, Zhao C, Bai Z, et al. Diagnosing *Balamuthia mandrillaris* amebic meningoencephalitis in a 64-year-old woman from the Southwest of China. Parasites Hosts Dis. 2023;61:183–93. PubMed https://doi.org/10.3347/PHD.23039

18. Liang Y, Wang W. A Balamuthia amoebic encephalitis survivor in China, and literature review. Diagn Microbiol Infect Dis. 2025;111:116698. PubMed https://doi.org/10.1016/j.diagmicrobio.2025.116698

19. Wang WN, Liang YM, Wang YN, Zhang S. Balamuthia mandrillaris amebic encephalitis: report of a case [in Chinese]. Zhonghua Bing Li Xue Za Zhi. 2024;53:1284–6. PubMed

20. Xu H, Wang D, Cui K, Wan R, Chi Q, Wu T. 18F-FDG PET/CT findings in fatal *Balamuthia Mandrillaris* encephalitis in brain stem: a case report. Radiol Case Rep. 2024;19:1851–4. PubMed https://doi.org/10.1016/j.radcr.2024.02.021

21. Guan W, Zhao H, Shao S, Zheng N. *Balamuthia mandrillaris* meningoencephalitis: a case report and literature review [in Chinese]. Journal of Shandong First Medical University and Shandong Academy of Medical Sciences. 2024;45:412–4.

22. Li Z, Li W, Li Y, Ma F, Li G. A case report of *Balamuthia mandrillaris* encephalitis. Heliyon. 2024;10:e26905. PubMed https://doi.org/10.1016/j.heliyon.2024.e26905

23. Zhang C, Xue M. *Balamuthia mandrillaris* amoebic encephalitis: a case report [in Chinese]. Journal of Apoplexy and Nervous Diseases. 2024;41:1140–1.

24. Qin S, Lu X, Li L, Huang D. Nursing care in intensive care unit of a patient infected with *Balamuthia mandrillaris* after renal transplantation: a case report. Transplant Proc. 2024;56:1183–7. PubMed https://doi.org/10.1016/j.transproceed.2024.02.019

25. Chen L, Hu J, Zhang T, Guo J. *Balamuthia mandrillaris* encephalitis: a case report and literature review [in Chinese]. Gansu Medical Journal. 2024;43:861–4.

26. Qin L, Xiang Y, Wu Z, Zhang H, Wu X, Chen Q. Metagenomic next-generation sequencing for diagnosis of fatal *Balamuthia* amoebic encephalitis. Infect Genet Evol. 2024;119:105570. PubMed https://doi.org/10.1016/j.meegid.2024.105570

27. Zheng Z, Chen F, Qin L, Lu A, Xu H, Zhao M, et al. Application of ventriculoscopy in granulomatous amoebic encephalitis: a case report in China and literature review. Front Med (Lausanne). 2024;11:1431225. PubMed https://doi.org/10.3389/fmed.2024.1431225

28. Li D. Report of one case of *Balamuthia mandrillaris* encephalitis in human [in Chinese]. Chin J Zoonoses. 2024;40:696–700.

29. Wang S, Zhang Y, Yan M, Wu Y, Li Y, Yue X, et al. *Balamuthia mandrillaris* amoebic encephalitis: a case report [in Chinese]. Chinese Journal of Infection and Chemotherapy. 2024;24:85–8.

30. Fan J, Ha C, Xu D, Wang X, Zhou S, Dong F. *Balamuthia mandrillaris* Encephalitis: a case report [in Chinese]. Radiol Prat. 2024;39:1271–2.

31. Chen Y, Peng L, Tian H, Xu S. Nursing Care of a Case of Baboon *Balamuthia* Amoebic Encephaliti [in Chinese]. Journal of Chinese and Foreign Medicine and Pharmacy Research. 2024;3:129–31.

32. Zhao W, Fang L, Tian Z, Yang Y, Sun H, Wang Y, et al. *Balamuthia mandrillaris* Encephalitis: a case report [in Chinese]. J Clin Neurol. 2025;38:57–9.

33. Chen Y, Zhang Q, Xie Z, Liu S, Luo S. *Balamuthia mandrillaris* amoebic encephalitis:A case report and literature review [in Chinese]. Journal of Apoplexy and Nervous Diseases. 2025;42:361–4.

34. Liu H, Zhong X. *Balamuthia mandrillaris* Encephalitis in a child: a case report [in Chinese]. Journal of Gannan Medical University. 2025;45:255–6+300.

35. Chen T, Jin D, Ye H. *Balamuthia mandrillaris* amebic encephalitis in children: a case report [in Chinese]. Zhongguo Xue Xi Chong Bing Fang Zhi Za Zhi. 2025;37:447–50. PubMed

36. Xiong Y, Lou X. *Balamuthia mandrillaris* encephalitis post–raw beef ingestion. Ann Neurol. 2025;97:384–5. PubMed https://doi.org/10.1002/ana.27082

37. Liang Y, Liu Y, Chen Z, Sun J, Zhang X, Wang Y. *Balamuthia* amoebic encephalitis directly causing intracranial infection: a case report. Radiol Case Rep. 2025;20:2820–4. PubMed https://doi.org/10.1016/j.radcr.2025.02.035

38. Mei J, Sheng F, Zhang C, Chen X. Imaging monitoring of Balamuthia granulomatous amoebic encephalitis. Clin Neurol Neurosurg. 2025;254:108917. PubMed https://doi.org/10.1016/j.clineuro.2025.108917

39. Teng Z, Liu L, Chen T, Liang J, Yao X, Zhao N, et al. A novel *Balamuthia* lineage causing fatal granulomatous amebic encephalitis in an immunocompetent infant. Int J Infect Dis. 2025;161:108063. PubMed https://doi.org/10.1016/j.ijid.2025.108063
